# Educational differences in long-term care use in Sweden during the last two years of life

**DOI:** 10.1177/14034948211043658

**Published:** 2021-09-30

**Authors:** Susanne Kelfve, Jonas W. Wastesson, Bettina Meinow

**Affiliations:** 1 Linköping University, Department of Culture and Society, Division Ageing and Social Change and Division of Social Work, Sweden; 2 Aging Research Center, Karolinska Institutet and Stockholm University, Sweden; 3 Karolinska Institutet, Department of Medical Epidemiology and Biostatistics, Sweden; 4 Stockholm Gerontology Research Center, Sweden

**Keywords:** Elder care, level of education, sex, residential care, home-help services, register data, end of life

## Abstract

*Background:* In old age, many people experience a period of functional decline and require long-term care. Sweden has a universal largely tax-financed health and social care system that is used by all societal groups. However, few studies have investigated if educational groups use publicly paid long-term care equitably. The aim of this study was to explore educational differences in the use of long-term care, including both home care and institutional care, during the last two years of life in Sweden. *Methods:* We used linked register data on mortality and long-term care use, including all adults aged ⩾67 years who died in Sweden in November 2015 (*N*=6329). We used zero-inflated negative binomial regression models to analyse the number of months with long-term care by educational level, both crude and adjusted for age at death and cohabitation status. Men and women were analysed separately. *Results:* People with tertiary education died more commonly without using any long-term care compared to primary educated people (28.0% vs. 18.6%; *p*<0.001). In the adjusted model, educational differences in the estimated number of months with long-term care disappeared among men but remained significant among women (primary educated: odds ratio=17.3 (confidence interval 16.8–17.7); tertiary educated: odds ratio=15.8 (confidence interval 14.8–16.8)). ***Conclusions:* Older adults spend considerable time in their last two years of life with long-term care. Only minor educational differences in long-term care use remained after adjustment for cohabitation status and age at death. This suggest that Sweden’s publicly financed long-term system achieves relatively equitable use of long-term care at the end of life.**

## Introduction

Most deaths in low-mortality countries occur around 85–90 years of age [1,2] and are preceded by a period of functional decline and need for long-term care [3,4]. As the large cohorts born in the 1940s are about to pass the age of 80 during the next decade, this will increase the pressure on the long-term care system in Sweden and in other countries with large Baby Boom generations [5].

The length and timing of the period with health and functional limitations at the end of life differ between societal groups. Many studies have shown that socio-economic differences in health prevail into old age. Higher education is positively associated with both life expectancy and years in good health [6–10]. Less is known about how this is reflected in the use of long-term care among different educational groups. A study conducted in the UK, Italy, Belgium and the Netherlands found a socio-economic gradient in the use of formal and informal home-care services for older people, which was largely explained by age, health and marital status [11]. Swedish studies have also suggested educational differences in care use. High education was associated with a lower probability of institutional care at death, accompanied with a higher probability of dying in hospital, even after adjusting for sex, age and health [12]. People with higher education have also been reported to buy more private services, such as house cleaning, in the course of declining public long-term care, while lower educated people are more likely to receive more informal care [13].

Sweden has a universal largely tax-financed health and social care system. Adult children have no legal obligation to provide or finance care for their parents. Municipalities are responsible for providing long-term care, including home care and institutional care. Services are *needs* tested but not *means* tested, and such services are granted by municipal need assessors. Home care covers practical support with household chores and/or personal care (e.g. dressing, showering and toileting). Home care can be offered around the clock and may be complemented with health care in the home. Sweden employs an ‘aging in place’ policy, where extensive home help is prioritised before institutional care [14,15]. Individual income-related user fees are low, with a current cap of ~$225 per month, covering only 4–5% of the actual costs [15]. Family or household economic resources are not considered. Hence, long-term care is available for everyone who is considered to be in need, and is generally used by all societal groups, regardless of economic resources. Privately purchased help with personal care is almost non-existent in Sweden. However, since taxpayers of all ages are entitled to deduct 50% of the price of household services, people with a high income or with pensions sometimes choose privately paid household services over publicly financed home care for household chores.

There are several possible mechanisms that may lead to educational differences in long-term care use. On the one hand, higher education is generally associated with better health [6–10], and it could therefore be hypothesised that higher educated people use less long-term care than lower educated people. On the other hand, people with higher education tend to live longer, and we have previously reported that older age is associated with a longer period with long-term care [16]. This could imply a longer period with long-term care among higher educated people compared to lower educated people. None of these possible mechanisms underlying educational differences in long-term care use could be viewed as inequity (i.e. as unfair differences), since they could be due to different needs for care. However, another factor related to long-term care use may be that higher educated people, and their relatives, tend to have more resources, in terms of both the means and knowledge about different care solutions, which could affect both the type and amount of long-term care they use, including different use of privately purchased household services [13]. The goal of a well-functioning welfare state is equitable distribution of services, that is, to provide the same care to people with the same need for care. So far, few studies [12,13] have investigated the association between level of education and the use of public financed long-term care in Sweden. Given the Swedish welfare system, where long-term care is needs assessed, we hypothesised that the educational differences in long-term care use during the last years of life would be small or non-existent.

### Aim

In this study, we aimed to explore educational differences in the use of long-term care, including both home care and institutional care, during the last two years of life in Sweden. We also considered the role of sex, cohabitation status and age at death – factors that have been shown to be associated with long-term care use in Finland [17] and Sweden [18]. By focusing on the two last years of life, we aimed to limit influences of the association between education and health, that is, the expectation that people with a high education in general live longer in good health. Based on the assumption that time to death is a relatively good proxy of health and care need in old age [19], we expected health and care needs to be more similar during the last two years of life, given the same age at death, compared to health and care needs between older people with different remaining lifetime.

## Methods

### Study population and data material

This was a retrospective cohort study based on Swedish register data covering all individuals aged ⩾67 years who died in Sweden in November 2015. In total, 6329 individuals were identified in the Cause of Death Register, and they were individually linked with retrospective information on long-term care use (from December 2013 until November 2015) in the Social Service Register (from the National Board of Health and Welfare). Information about the highest level of education, sex and cohabitation status come from Statistics Sweden. For more information about the study population and the register data, see Meinow et al. [16]. Of the 6329 individuals, we excluded those who immigrated after 30 November 2013 (*n*=8) and those with incomplete information on long-term care use (*n*=373) or education (*n*=143). The final sample therefore consisted of 5805 individuals. Ethical approval for record linkage of the Swedish register data was obtained from the Stockholm Regional Ethical Review Board (Dnr 2016/1001-31/4).

### Outcome measures

We measured long-term care use during the 24-month study period and created four different variables: no long-term care (neither home-help services nor institutional care), no institutional care, number of months with long-term care (either home care or institutional care) and number of months with institutional care.

### Covariates

We coded the highest level of education into primary education or less (compulsory education, which in Sweden corresponds to a maximum of nine years), secondary education (all education between compulsory and tertiary education, often two to four years) and tertiary education (at least one year of university education). We coded cohabitation status as living with a partner or not at the end of 2014, which corresponds to the latest time point that we had this information from Statistics Sweden.

### Statistical analyses

We described the study population by summarising educational differences in use of long-term care and cohabitation status as percentages, and age at death as means with standard deviations. We predicted the number of months in long-term care by zero-inflated negative binomial regression models to account for the many non-users and the skewed distribution of the number of months with long-term care. Zero-inflated regressions consist of a logistic part, estimating the probability of non-use (0/1), and a Poisson part, estimating the number of months with long-term care (1–24 months) among users. Average marginal effects were calculated to estimate the average number of months with long-term care in total for each analysed group. We predicted the number of months in long-term care crude, and adjusted by age at death and cohabitation status, by education. The analyses were performed for men and women separately. We also added covariates one by one to disentangle the separate effect of cohabitation and age at death. All analyses were performed with Stata v14 (StataCorp, College Station, TX).

## Results

Compared to primary educated people, those with higher education died more frequently without any long-term care (primary=18.6%, secondary=24.2%; *p*<0.001, tertiary=28.0%; *p*<0.001) as well as without institutional care (primary=57.8%, secondary=64.2%; *p*=0.025, tertiary=70.0%; *p*=0.002). These differences were more pronounced among women than among men (Table I). Not using any long-term care before death was most common among cohabiting women with tertiary education (42.6%) and least common among women living alone with primary education (9.3%; not shown).

**Table I.** Use of long-term care (including home care and institutional care) during the last two years of life, cohabitation status and age at death among people who died in November 2015 by sex and education.

All	Total		Primary education	Secondary education	*p*-Value^a^	Tertiary education	*p-*value^a^
	*N*=5805		*N*=3022	*N*=1966		*N*=817	
	*n*	%	%	%		%	
Cohabiting	1958	33.7	30.2	35.2	*0.008*	43.6	*<0.001*
No long-term care	1267	21.8	18.6	24.2	*<0.001*	28.0	*<0.001*
No institutional care	3580	61.7	57.8	64.2	*0.025*	70.0	*0.002*
Months in long-term care, *M* (*SD*)	14.8 (10.5)		15.8 (10.2)	14.0 (10.6)	*<0.001*	12.8 (10.9)	*<0.001*
Age at death, *M* (*SD*)	83.8 (8.3)		85.2 (8.0)	82.4 (8.4)	*<0.001*	81.8 (8.4)	*<0.001*
Men	*N*=2753		*N*=1326	*N*=986		*N*=441	
		*n*	*%*	%	%		%	
Cohabiting	1336	48.5	46.6	47.0	0.867	57.8	<0.001
No long-term care	791	28.7	26.7	29.9	0.088	32.2	0.026
No institutional care	1932	70.2	67.3	71.7	0.022	75.5	0.001
Months in long-term care, *M* (*SD*)	12.5 (10.7)		13.2 (10.7)	12.1 (10.6)	0.011	11.5 (10.8)	0.003
Age at death, *M* (*SD*)	82.1 (8.1)		83.1 (7.9)	81.3 (8.2)	<0.001	81.2 (8.1)	0.679
Women	*N*=3052		*N*=1696	*N*=980		*N*=376	
	*n*	%	%	%		%	
Cohabiting	622	20.4	17.3	23.3	<0.001	26.9	<0.001
No long-term care	476	15.6	12.3	18.5	<0.001	23.1	<0.001
No institution	1648	54.0	50.4	56.6	0.002	63.6	<0.001
Months in long-term care, *M* (*SD*)	17.0 (9.9)		18.2 (9.2)	15.9 (10.3)	<0.001	14.2 (10.8)	<0.001
Age at death, *M* (*SD*)	85.3 (8.2)		86.9 (7.7)	83.6 (8.4)	<0.001	82.5 (8.6)	<0.001

a *p*-Values refer to *t*-test (continuous outcomes) or chi-square test (categorical outcomes) compared to the primary educated group.

*SD*: standard deviation.

The crude regression models in Table II (Model 1) confirmed the negative association between educational level and long-term care use. Among women, high education was associated with higher odds of not having used any long-term care (secondary: odds ratio (OR)=1.62, 95% confidence interval (CI) 1.27–1.97; tertiary: OR=2.15, 95% CI 1.55–2.76). Among female long-term care users, results for high education showed lower estimates of the number of months with long-term care (secondary: OR=0.94, 95% CI 0.90–0.98; tertiary: OR=0.89, 95% CI 0.84–0.95). In total, the average number of months with long-term care during the last two years of life was estimated to be 18.17 months (95% CI 17.63–18.72) for primary educated women and 14.22 months (95% CI 13.11–15.33) for women with tertiary education. Most of the educational differences in long-term care among women were explained by cohabitation and age at death (Model 2). The predicted number of months with long-term care remained slightly higher for women with primary education compared to those with tertiary education (OR=17.27, 95% CI 16.79–17.74 vs. OR=15.83, 95% CI 14.85–16.80).

**Table II.** Estimated use of long-term care (including home care and institutional care) during the last two years of life among women and men by educational level, crude (Model 1) and adjusted for age at death and cohabitation status (Model 2).

Estimate of non-use	Women *N*=3052 Users=2576 Non-users=476				Men *N*=2753 Users=1962 Non-users=791			
	Model 1^a^		Model 2^b^		Model 1^a^		Model 2^b^	
	OR	95% CI	OR	95% CI	OR	95% CI	OR	95% CI
*Educational level*								
Primary	Ref.		Ref.		Ref.		Ref.	
Secondary	1.62	1.27–1.97	1.08	0.82–1.35	1.17	0.96–1.39	1.01	0.80–1.21
Tertiary	2.15	1.55–2.76	1.29	0.89–1.70	1.30	1.00–1.61	1.03	0.76–1.30
Estimate of number of months among users	RR	95% CI	RR	95% CI	RR	95% CI	RR	95% CI
*Educational level*								
Primary	Ref.		Ref.		Ref.		Ref.	
Secondary	0.94	0.90–0.98	0.99	0.95–1.02	0.96	0.90–1.02	0.99	0.93–1.05
Tertiary	0.89	0.84–0.95	0.94	0.89–1.00	0.94	0.87–1.02	0.98	0.91–1.06
Estimated average months in total		95% CI		95% CI		95% CI		95% CI
*Educational level*								
Primary	18.17	17.63–18.72	17.27	16.79–17.74	13.17	12.49–13.84	12.61	12.02–13.21
Secondary	15.92	15.22–16.63	16.88	16.26–17.51	12.07	11.32–12.82	12.46	11.76–13.15
Tertiary	14.22	13.11–15.33	15.83	14.85–16.80	11.51	10.40–12.62	12.33	11.30–13.37

a Model 1: bivariate associations.

b Model 2: controlled for age at death and cohabitation status.

CI: confidence interval; OR: odds ratio; RR: rate ratio; Ref.: reference category.

For men, the average number of months in long-term care and the educational differences in long-term care use were lower compared to women. The average number of months with long-term care among primary educated men was estimated to be 13.17 months (95% CI 12.49–13.84) compared to 11.51 months (95% CI 10.40–12.62) among men with tertiary education. In contrast to women, for men, all educational differences in long-term care use disappeared when adjusting for cohabitation and age at death (Model 2).

Analyses of the use of institutional care (Table III) showed a similar association with level of education as shown for any long-term care. Crude models (Model 1) showed that the average number of months in institutional care during the last two years of life among primary educated women was estimated to be 9.07 months (95% CI 8.52–9.62) compared to 6.10 months (95% CI 5.10–7.09) among women with tertiary education. Corresponding numbers for men were 5.62 months (95% CI 5.08–6.16) for primary educated and 4.11 months (95% CI 3.29–4.93) for tertiary educated.

**Table III.** Estimated use of institutional care during the last two years of life among women and men by educational level, crude (Model 1) and adjusted for age at death and cohabitation status (Model 2).

*Estimate of non-use*	Women *N*=3052 Users=1404 Non-users=1648				Men *N*=2753 Users =821 Non-users =1932			
	Model 1^a^		Model 2^b^		Model 1^a^		Model 2^b^	
	OR	95% CI	OR	95% CI	OR	95% CI	OR	95% CI
*Educational level*								
Primary	Ref.		Ref.		Ref.		Ref.	
Secondary	1.29	1.08–1.49	0.97	0.80–1.13	1.23	1.01–1.45	1.09	0.88–1.30
Tertiary	1.72	1.32–2.12	1.20	0.90–1.51	1.50	1.13–1.87	1.24	0.91–1.56
Estimate of number of months among users	RR	95% CI	RR	95% CI	RR	95% CI	RR	95% CI
*Educational level*								
Primary	Ref.		Ref.		Ref.		Ref.	
Secondary	0.98	0.92–1.04	1.00	0.93–1.06	0.95	0.86–1.03	0.96	0.87–1.05
Tertiary	0.92	0.82–1.01	0.95	0.85–1.04	0.98	0.85–1.10	1.00	0.87–1.13
Estimated average months in total		95% CI		95% CI		95% CI		95% CI
*Educational level*								
Primary	9.07	8.52–9.62	8.37	7.88–8.86	5.62	5.08–6.16	5.27	4.78–5.76
Secondary	7.76	7.07–8.44	8.47	7.79–9.16	4.60	4.04–5.16	4.81	4.26–5.36
Tertiary	6.10	5.10–7.09	7.28	6.21–8.34	4.11	3.29–4.93	4.63	3.76–5.49

a Model 1: bivariate associations.

b Model 2: controlled for age at death and cohabitation status.

As with any form of long-term care use, the educational differences in institutional care among men could be explained by cohabitation and age at death (Model 2). Among women, the predicted number of months in institutional care differed by less than a month between women with primary education and tertiary education after adjustment (OR=8.37, 95% CI 7.88–8.86 vs. OR=7.28, 95% CI 6.21–8.34).

Adding covariates one by one (not shown) demonstrated that age at death was the main contributing factor to the educational differences in long-term care use, among both men and women. However, cohabitation was associated with a shorter duration of institutional care among men.

## Discussion

Based on Swedish register data, comprising all people aged ⩾67 years who died in November 2015, we showed that people with higher education used less long-term care during the last two years of life than people with lower education did, and they died more often without using any long-term care. However, results also showed that after adjustment for age at death and cohabitation, no educational differences in long-term care use remained among men, and most differences among women disappeared. This means that given the same age at death and the same cohabitation status, there are very small educational differences in long-term care use in Sweden during the last two years of life.

A social gradient in socio-economic factors and the use of long-term care has been shown in a study of four European countries: the UK, Italy, Belgium and the Netherlands [11]. Notably, the negative association between socio-economic factors and formal care was to large extent explained by age, health and marital status. However, the authors stated that the mechanisms of the association between socio-economic factors and care use seemed to vary between culture and welfare systems in the included countries [11]. Although it is difficult to compare results from different countries, our results also showed that age at death was the main contributing factor behind educational differences in the use of long-term care.

When interpreting our results, it is important to have the retrospective nature of the data in mind. In general, people with high education tend to live longer than those with low education. Also, higher education is more common in later-born cohorts. In our analyses, all people died in 2015. Hence, by design, people dying at a younger age belonged to more well-educated cohorts. This is the reason why the mean age at death is four years lower among people with high education compared to those with low education. However, we do not consider a prospective design to be an option for this study, as it would have required a very long follow-up period, allowing for people in different cohorts both to enter long-term care and to die.

This retrospective design may also have contributed to the large educational differences in the crude analyses. Young decedents, belonging to later-born and more well-educated cohorts, may to a greater extent die from fast-acting incurable diseases, for example specific types of cancer that are related to a rapid terminal decline, and thus use less long-term care. Older decedents, on the other hand, are more likely to die from degenerative diseases associated with a longer period of long-term care [20]. The differences in cause of death may partly explain the large educational differences in the crude analyses. Consequently, we found small or no differences between educational groups when age at death was taken into account.

Overall, in line with previous findings, women used more long-term care than men did during the last two years of life [21]. Results also showed that age at death was the main contributing factor for the educational differences in long-term care use among both men and women. However, among men, cohabitation was associated with a shorter duration of institutional care. This suggests that the availability of a cohabiting partner tends to postpone the move to an institution more among men than among women. This highlights that other factors than care needs, such as access to informal care, may also influence the use of publicly paid long-term care. The use of privately paid household services with tax deduction could also contribute to a later entrance into long-term care, especially among people with a high income or pensions. Moreover, privately paid help with personal care is almost non-existent in Sweden. It has also been shown that those with informal care due to substantial care needs tend to use formal care, implying a complementary model rather than a model of substitution [22], which has also been shown previously within the Swedish universal welfare system [23]. However, we do not have information about informal or privately paid help.

In the coming years, increasing pressure on the long-term care system can be expected as the large cohorts born in the 1940s have begun to pass the age of 80 [5]. If this demographic process is not compensated economically, it is likely that we will see a narrower definition of public responsibility for long-term care. This may result in widening inequalities in long-term care use as better-off older people tend to turn to privately purchased care, while other groups are left to alternative solutions, such as informal care [13].

The few studies that have analysed educational differences in long-term care use in Sweden have focused on place of death [12] or have analysed long-term care use based on cross-sectional data [13]. This study adds to previous knowledge by including longitudinal data over two years and covering both home-care services and institutional care. The main strength of our study is the nationwide coverage and the lack of non-participation bias. Although we lacked information about health-related need indicators, we minimised this limitation by focusing the analyses on the last two years of life. In addition, long-term care in Sweden is strictly needs assessed, which means that not all people who would be eligible apply for and use services, but all those who do have gone through a needs assessment. There is no national register information on individuals who applied for but were not granted long-term care in Sweden. Disabilities with activities of daily living and cognitive impairment (in particular regarding institutional care) have been found to be the most important predictors for the amount of long-term care use in Sweden [24]. Another limitation is that our analyses do not include the cause of death, which could further elucidate the association between education and long-term care use.

Based on these limitations, we need to be careful when interpreting the results. Although we found relatively equal use of publicly financed long-term care between educational groups, we cannot be certain that long-term care is distributed equitably (i.e. according to need). It is possible that people with different levels of education die from different diseases, although they die at the same age. If some diseases require more care and at the same time are more common in one educational group, equal distribution of care between educational groups implies an unequal distribution. Hence, we cannot rule out that people with high education use their resources to obtain similar care services as those with primary education, although they do not have the same care needs.

It should also be mentioned that the importance of education for health and living conditions might change over time and hence between different cohorts. In earlier-born cohorts, educational level is not as strongly associated with cognitive, economic and social resources as in later-born cohorts [25], and this may in particular be the case for women in older cohorts who often left school at the minimum age with no qualifications [26]. Hence, the association between education on health and the need for long-term care are probably stronger for later-born cohorts.

## Conclusions

This study showed that higher levels of education were associated with a higher probability of dying without long-term care as well as a shorter period with long-term care. However, the lower use of long-term care among high-educated people could mainly be explained by compositional factors. Those who died at younger ages (i.e. younger cohorts) not only used less long-term care [16], they were also more often highly educated. Adjusted for age at death and cohabitation status, most educational differences in long-term care use disappeared. This means that in Sweden, different educational groups use publicly financed long-term care fairly equitably during the last two years of life. With an increasing demand for long-term care, it is important to continue monitoring the use of long-term care within a publicly financed system.
